# Large vessel involvement by IgG4-related disease

**DOI:** 10.1097/MD.0000000000003344

**Published:** 2016-07-18

**Authors:** Cory A. Perugino, Zachary S. Wallace, Nandini Meyersohn, George Oliveira, James R. Stone, John H. Stone

**Affiliations:** Massachusetts General Hospital, Boston, MA.

**Keywords:** aortitis, coronary artery aneurysm, IgG4-related disease (IgG4-RD), inflammatory aortic aneurysm, large vessel vasculitis, periaortitis, pulmonary artery aneurysm, retroperitoneal fibrosis

## Abstract

**Objectives:**

IgG4-related disease (IgG4-RD) is an immune-mediated fibroinflammatory condition that can affect multiple organs and lead to tumefactive, tissue-destructive lesions. Reports have described inflammatory aortitis and periaortitis, the latter in the setting of retroperitoneal fibrosis (RPF), but have not distinguished adequately between these 2 manifestations. The frequency, radiologic features, and response of vascular complications to B cell depletion remain poorly defined. We describe the clinical features, radiology findings, and treatment response in a cohort of 36 patients with IgG4-RD affecting large blood vessels.

**Methods:**

Clinical records of all patients diagnosed with IgG4-RD in our center were reviewed. All radiologic studies were reviewed. We distinguished between primary large blood vessel inflammation and secondary vascular involvement. Primary involvement was defined as inflammation in the blood vessel wall as a principal focus of disease. Secondary vascular involvement was defined as disease caused by the effects of adjacent inflammation on the blood vessel wall.

**Results:**

Of the 160 IgG4-RD patients in this cohort, 36 (22.5%) had large-vessel involvement. The mean age at disease onset of the patients with large-vessel IgG4-RD was 54.6 years. Twenty-eight patients (78%) were male and 8 (22%) were female. Thirteen patients (36%) had primary IgG4-related vasculitis and aortitis with aneurysm formation comprised the most common manifestation. This affected 5.6% of the entire IgG4-RD cohort and was observed in the thoracic aorta in 8 patients, the abdominal aorta in 4, and both the thoracic and abdominal aorta in 3. Three of these aneurysms were complicated by aortic dissection or contained perforation. Periaortitis secondary to RPF accounted for 27 of 29 patients (93%) of secondary vascular involvement by IgG4-RD. Only 5 patients demonstrated evidence of both primary and secondary blood vessel involvement. Of those treated with rituximab, a majority responded positively.

**Conclusions:**

IgG4-RD is a distinctive, unique, and treatable cause of large-vessel vasculitis. It can also involve blood vessels secondary to perivascular tumefactive lesions. The most common manifestation of IgG4-related vasculitis is aortitis with aneurysm formation. The most common secondary vascular manifestation is periaortitis with relative sparing of the aortic wall. Both primary vasculitis and secondary vascular involvement respond well to B cell depletion therapy.

## 1 Introduction

IgG4-related disease (IgG4-RD) is an immune-mediated fibroinflammatory condition that can affect multiple organs and lead to tumefactive, tissue-destructive lesions.^[1]^ Histopathological examination of these lesions reveals both inflammatory elements in the form of a lymphoplasmacytic infiltrate, often accompanied by a mild tissue eosinophilia, and a fibrotic component characterized by “storiform” fibrosis. Immunohistochemistry studies reveal a preponderance of IgG4-positive plasma cells. Recognition of this condition as a distinct entity stemmed from the investigation of autoimmune pancreatitis, which was ultimately found to be part of a larger systemic process.^[2]^ Over the last decade, IgG4-RD has been reported to involve nearly every organ system.^[3]^

Microscopic vascular findings of IgG4-RD—namely, obliterative phlebitis, and obliterative arteritis—have been recognized since 2003.^[4]^ It was not until 2008, however, that large-vessel involvement by IgG4-RD was recognized in the form of “inflammatory abdominal aortic aneurysm.”^[5]^ A variety of other macrovascular manifestations of IgG4-RD have been described since then, including coronary periarteritis,^[6]^ inflammatory thoracic aortic aneurysms,^[7]^ and periaortitis/arteritis in the setting of retroperitoneal fibrosis (RPF).^[8,9]^ Such vascular manifestations can be associated with morbidity and mortality in the form of aortic dissection^[5]^ and sudden cardiac death.^[10]^ IgG4-RD was recognized as a potential cause of large-vessel vasculitis in the 2012 Chapel Hill Consensus Conference on Nomenclature of Vasculitides but remains a poorly understood entity [Jennette, 2013].^[11]^

Review of the literature suggests that 2 forms of large-vessel disease may exist in IgG4-RD: a primary vasculitis and a secondary form of vascular involvement characterized by periaortic or periarterial involvement. However, most literature on IgG4-related large-vessel involvement to date has not distinguished clearly between these 2 forms. We studied a cohort of 160 IgG4-RD patients whose disease features have been characterized carefully. Among these patients, 36 (22.5%) were recognized as having features of vascular involvement.

## 2 Methods

### 2.1 Cohort overview

This study was approved by the institutional review board of the Massachusetts General Hospital. All patients provided written consent to participate. The diagnosis of IgG4-RD hinges on the correlation of clinical findings with both specific histopathologic features and an increased number of IgG4+ plasma cells (or IgG4+/IgG+ ratio) in the affected tissue. The major histopathologic features include a dense lymphoplasmacytic infiltrate, obliterative phlebitis, and fibrosis that has a “storiform” (from the Latin storea, for “woven mat”) pattern in focal or diffuse areas. The presence of 2 of these features is highly suggestive of an IgG4-RD diagnosis.^[12]^ In contrast, a probable histologic diagnosis requires at least one of these histopathology features in conjunction with supportive clinical findings. The presence of nonobliterative phlebitis and mild to moderate degrees of tissue eosinophilia, both considered to be minor pathologic features, can strengthen the likelihood of IgG4-RD.^[12]^

In addition, a cutoff IgG4+ plasma cells per high-power field (hpf) ranging from 10 to 200 cells/hpf (depending on the biopsied organ) and an IgG4+/IgG+ ratio of at least 0.40 was considered essential to the histopathologic diagnosis. Because elevated IgG4+ plasma cells have been documented with other etiologies of aortitis, a density of 50 cells/400× hpf and an IgG4+/IgG+ ratio of at least 0.50 was required for cases relying on aortic tissue to establish the diagnosis. All of the above histologic definitions are in accordance with international consensus criteria on the pathology of IgG4-RD.^[12]^

Flow cytometry, performed on all samples at the time of patients’ presentations with active, untreated disease, was used to measure the absolute plasmablast count per milliliter by gating peripheral blood for CD19lowCD20−CD38+CD27+. Methods for collecting plasmablasts have been described.^[13]^ All serum IgG4 concentration measurements were performed with a serial dilution protocol to prevent the prozone phenomenon.^[14]^

Inflammatory vascular involvement was defined by objective radiologic or pathologic findings in all patients except 2, who were diagnosed on clinical grounds. Patient #1 was a 35-year-old man with a carotid occlusion and biopsy-proven IgG4-related RPF. Patient #4 involved an aortic dissection in a 32-year-old man with no atherosclerotic risk factors and a negative genetic evaluation for Ehlers–Danlos syndrome. He had a moderate serum IgG4 concentration elevation and the single slide available from the aortic surgery demonstrated a lymphoplasmacytic infiltrate.

### 2.2 Clinical data

Electronic records of all patients were reviewed. Data pertaining to demographics, comorbidities, clinical manifestations, organ involvement, laboratory studies, pathology, and treatment were included. Relevant comorbid conditions such as hypertension, hyperlipidemia, diabetes mellitus, chronic kidney disease, and arterial disease in the coronary, carotid, or extremity vessels were recorded. Laboratory data before and after rituximab administration (at 3 and 6 months) were also collected.

### 2.3 Radiographic data

All available vascular radiology data were reviewed and interpreted independently by 2 vascular radiologists. The radiology studies included computed tomography (CT) angiography, magnetic resonance (MR) angiography, and positron emission tomography (PET).

Primary IgG4-related vasculitis was defined by the presence of 1 or more of the following: vessel wall thickening; vessel wall enhancement on contrast study; or ^18^fluorodeoxyglucose (FDG) avidity within the blood vessel wall on PET. For the latter 2 features, the intensity of these findings were required to exceed that of typical atherosclerotic disease.

Secondary IgG4-related vascular involvement was defined by abnormal perivascular soft tissue with *minimal* vascular wall findings. The main radiologic findings were: enhancing soft tissue adjacent to or encasing the vessel; minimal vascular wall thickening or enhancement; ^18^FDG avidity in the perivascular region; and minimal luminal narrowing.

Patients with coronary artery involvement were defined by either abnormal FDG uptake on PET imaging or large coronary artery aneurysms with circumferential mural thickening without concurrent evidence of atherosclerotic plaque on coronary CT angiography. Carotid arteritis was defined clinically as explained above in Patient #1, histopathologically in another, and radiographically in a 3rd with both contrast enhancement and FDG avidity of the vessel wall. Iliac phlebitis was defined by the presence of abnormal T2 hyperintensity on MR imaging along the iliac veins and associated abnormal FDG uptake on PET imaging. The presence of aortic or common iliac arterial aneurysms as well as bilateral hydroureteronephrosis, ureteral displacement, and lymphadenopathy were also evaluated.

Abdominal aortic aneurysm was defined as a double oblique short-axis diameter of greater than 3.0 cm.^[15]^ Thoracic aortic aneurysm was defined as a double oblique short-axis diameter of greater than 3.8 cm in the ascending thoracic aorta and greater than 3.0 cm in the descending thoracic aorta.^[16]^ Common iliac artery aneurysm was defined as a double oblique short-axis diameter of greater than 1.5 cm. Radiologic improvement following treatment was determined by either a decrease in thickening of the blood vessel wall, perivascular soft tissue thickening, contrast enhancement, or FDG avidity.

### 2.4 Pathology data

Aortitis was defined as inflammation of the adventitia of the aorta with at least focal involvement of the media and or intima. By consensus criteria, a pathologic diagnosis of IgG4-related aortitis/periaortitis required greater than 50 IgG4+ plasma cells/400× hpf, with more than 50% of the plasma cells staining for IgG4. The presence of storiform fibrosis, obliterative adventitial phlebitis, and eosinophil infiltration was also determined.

### 2.5 Statistics

Clinical variables were compared between the primary vasculitis and secondary IgG4-related vasculopathy groups. These included laboratory parameters, age at disease onset, gender, number of organs involved and signs/symptoms attributed to vascular involvement. Differences in continuous, nonparametric variables (total IgG, IgG1, IgG4, CRP, and ESR) were compared using the Wilcoxon signed-rank test while parametric variables (age and number of organs involved) were compared using the *t* test. Differences in the distribution of categorical variables (gender and signs/symptoms of vascular disease) were compared with the Chi-square test. A 2-sided *P*-value of <0.05 was considered statistically significant for all tests.

## 3 Results

Among our cohort of 160 IgG4-RD patients, we identified large-vessel involvement in 36 (22.5%).

### 3.1 Clinical features

#### 3.1.1 Demographics

The demographic features of the large-vessel IgG4-RD patients are shown in Table 1. In this group, 28 (77%) were male. The mean age at onset of IgG4-RD was 54.6 ± 14 years. Twenty-eight patients (78%) had at least 1 established atherosclerotic risk factor including current or former cigarette smoking and diabetes mellitus (13 and 10 patients, respectively).

**Table 1:**
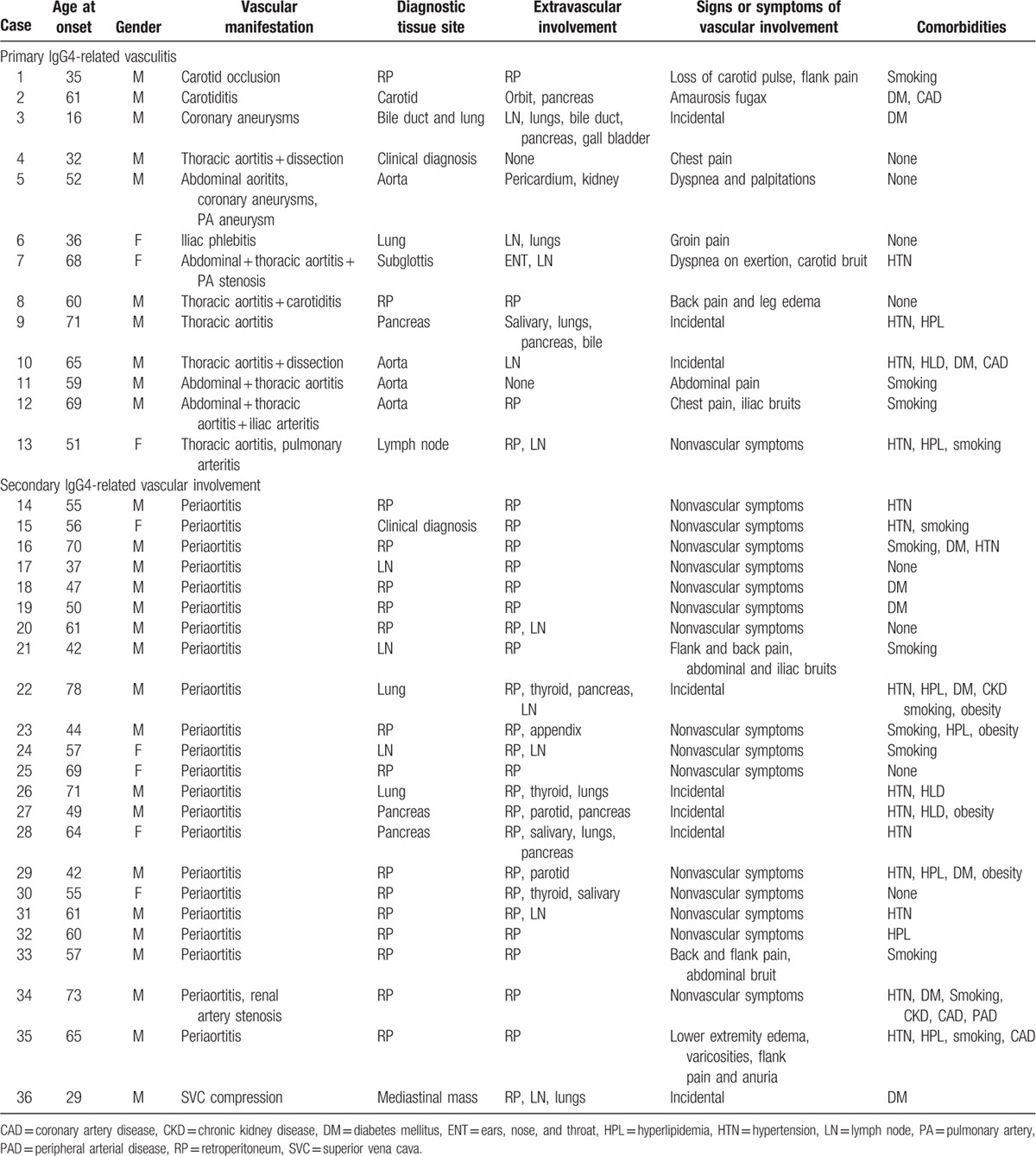
Demographic features and extravascular IgG4-related disease manifestations of the large-vessel disease patients.

#### 3.1.2 Clinical vignettes

We illustrate the spectrum of large-vessel disease in IgG4-RD by describing 6 representative patients in detail.

### 3.2 Patient #2: IgG4-related carotid arteritis

A 67-year-old man had a history of autoimmune pancreatitis, pericarditis, asthma, and peripheral eosinophilia of undetermined etiology. He had undergone a modified Whipple procedure for the erroneous diagnosis of pancreatic cancer before the correct diagnosis of lymphoplasmacytic sclerosing pancreatitis (now termed type 1 [IgG4-related] autoimmune pancreatitis) was established. He had become an insulin-requiring diabetic as a complication of this procedure. The patient was referred after developing swelling of the left parotid and bilateral lacrimal glands with associated left-sided proptosis.

CT imaging confirmed the clinical findings of bilateral enlarged lacrimal glands as well as enlargement of the left lateral rectus muscle. Bilateral lacrimal gland biopsies yielded a lymphoplasmacytic and eosinophilic cellular infiltrate, lymphoid follicles with germinal centers, and prominent periductal sclerosis within the lacrimal glands. Culture, microbial staining, and flow cytometry were negative for infectious and malignant etiologies. Immunohistochemistry showed 50 to 100 IgG4+ plasma cells/hpf, with >90% of all plasma cells staining for IgG4. A diagnosis of IgG4-related dacryoadenitis was made. Because of the patient's diabetes mellitus, he was treated with rituximab (1000 mg × 2 doses). By 3 months, the patient's proptosis, parotid gland swelling, and long-term nasal congestion had resolved completely and his serum IgG4 concentration had decreased from 1310 to 226 mg/dL. Repeat imaging showed slight improvement of the left orbital findings, resolution of the right orbital findings, and improved paranasal sinus mucosal thickening. Five months following induction, proptosis returned and radiologic findings worsened, prompting another course of rituximab, followed again by clinical and radiologic improvement.

**Figure 1:**
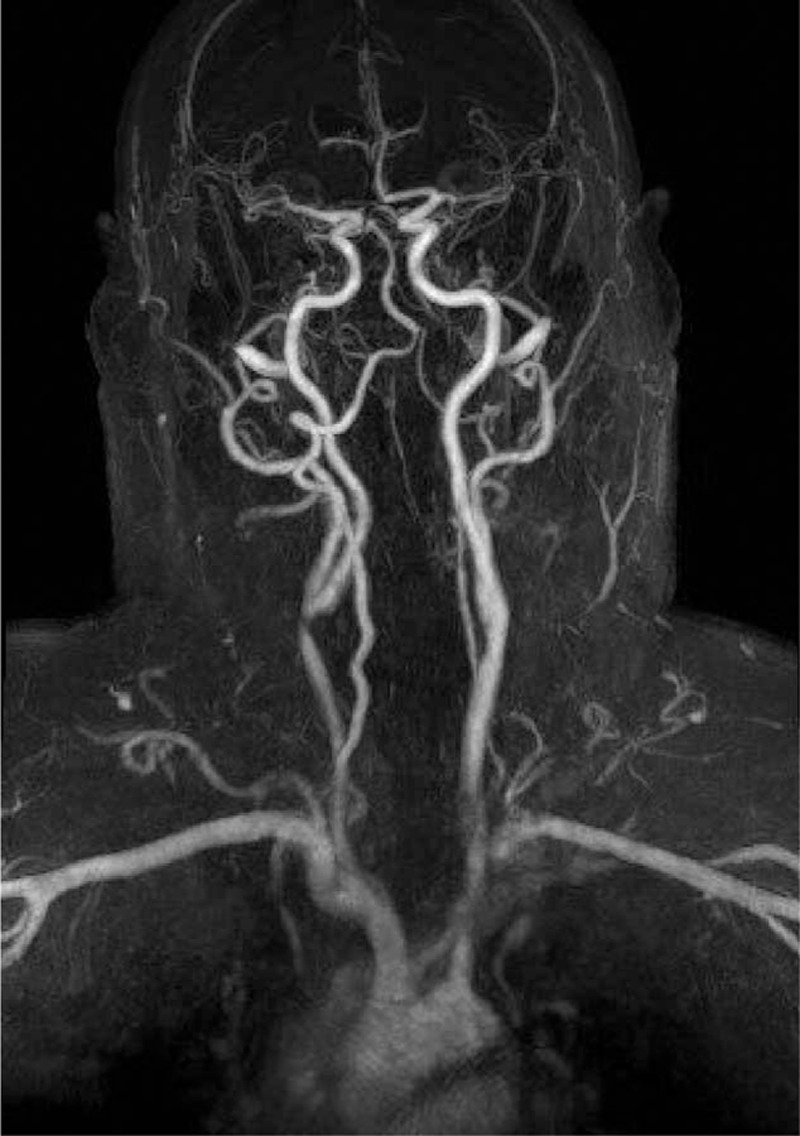
Magnetic resonance angiography with moderate to severe stenosis of the right common carotid artery.

**Figure 2:**
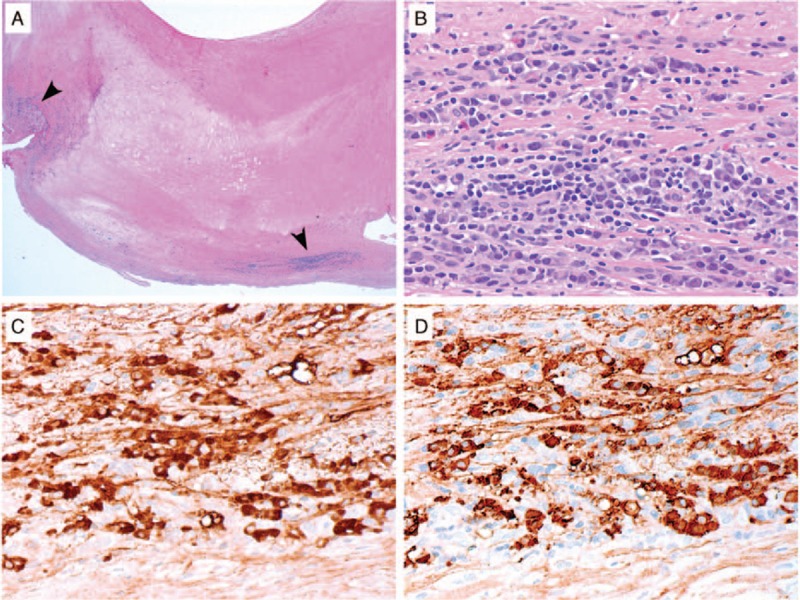
IgG4-related disease in a carotid artery atherosclerotic plaque. (A) Depicted is a low power (20× magnification) histologic image showing the carotid endarterectomy specimen from Case 2 with regions of dense inflammatory cell infiltration in the periphery (arrowheads). (B) At higher power (400× magnification) in these regions, there is a lymphoplasmacytic infiltrate with occasional eosinophils. By immunohistochemistry for IgG (C) and IgG4 (D), the majority of the IgG+ plasma cells expressed IgG4 (both 400× magnification).

Six months after the second rituximab course, the patient presented with recurrent episodes of right-sided amaurosis fugax. A magnetic resonance angiography (MRA) revealed moderate to severe stenosis of the right common carotid artery (Fig. 1). A carotid endarterectomy was undertaken without event, following which the amaurosis fugax resolved. Pathological examination of the carotid artery revealed a complex atherosclerotic plaque with a focal lymphoplasmacytic infiltrate and occasional eosinophils. More than 50% of the plasma cells within the lesion stained for IgG4 (Fig. 2) compatible with focal involvement by IgG4-RD. The patient underwent a third course of rituximab and it was subsequently decided to treat him with 1 g of rituximab every 3 months as maintenance therapy. On this regimen, his serum IgG4 has normalized and he has not experienced subsequent events related to IgG4-RD.

### 3.3 Patient #5: IgG4-related coronary artery aneurysms

**Figure 3:**
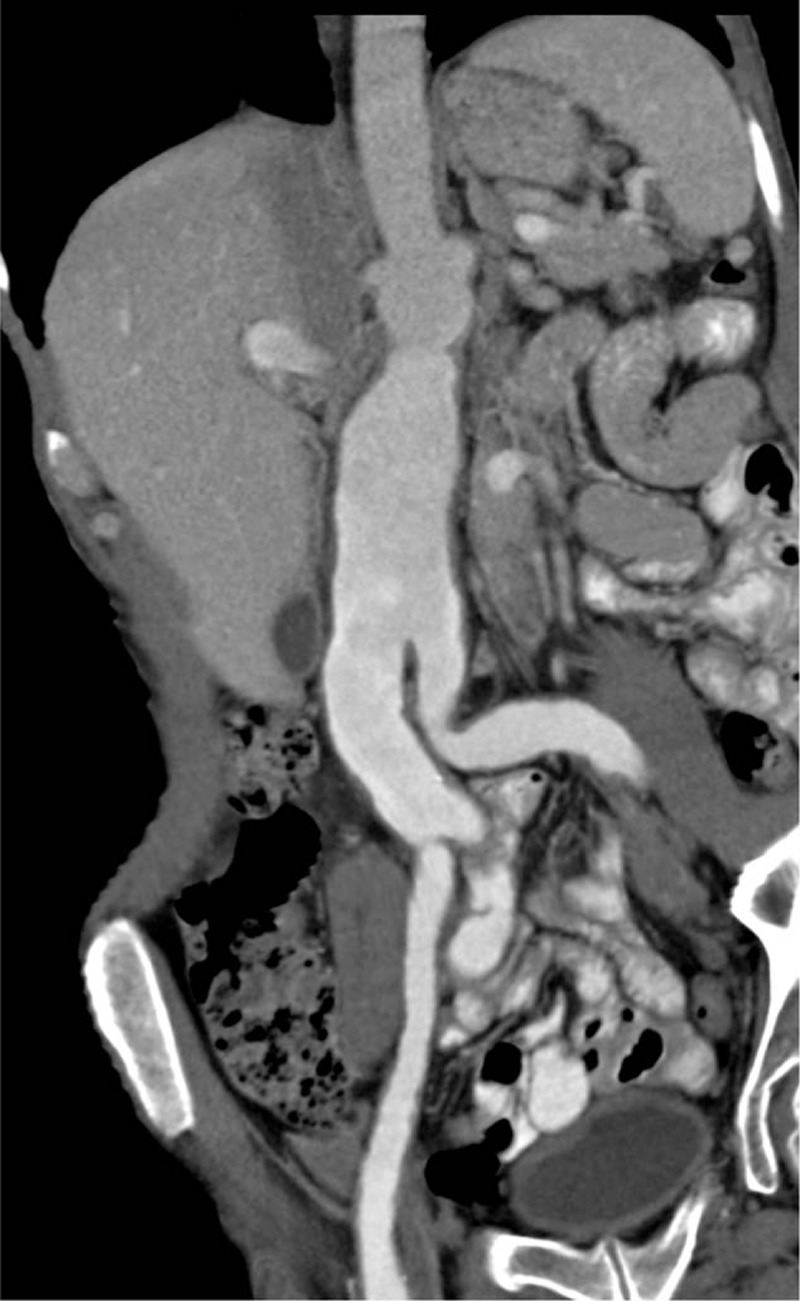
CT scan of the abdomen showing diffuse, nonatherosclerotic arterial disease with aneurysmal dilatation of the abdominal aorta and common iliac arteries.

**Figure 4:**
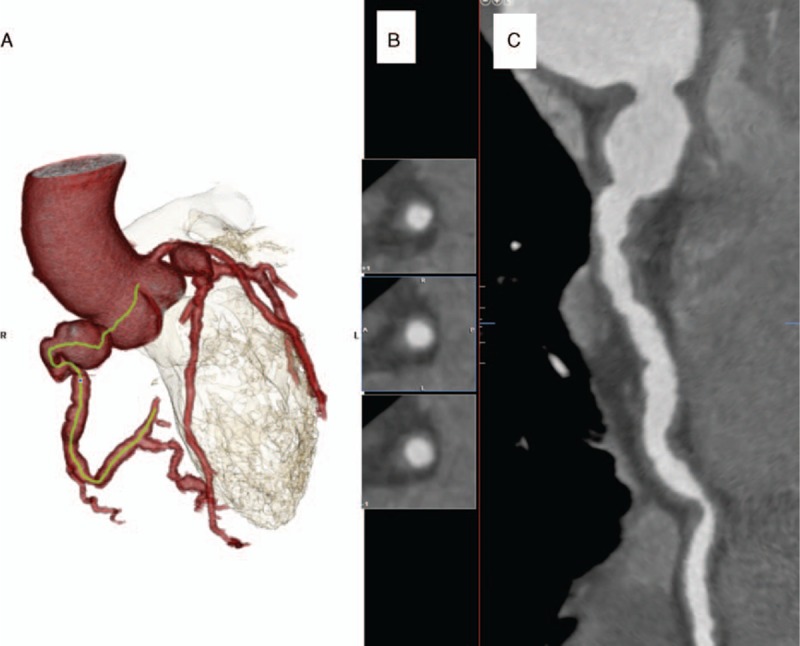
Right coronary artery and posterior descending artery with diffuse aneurysmal dilatation, circumferential mural thickening, and contrast enhancement as seen by 3D reconstruction (A), cross-sectional (B), and coronal (C) views.

A 54-year-old man with a history of non-Hodgkin lymphoma treated with rituximab + CHOP (cyclophosphamide/hydroxyurea/oncovin/prednisone) therapy 7 years before presentation experienced recurrent episodes of cardiogenic pulmonary edema, a non-ST segment myocardial infarction, and progressive kidney disease with proteinuria. Renal ultrasound examination revealed an incidental abdominal aortic aneurysm and bilateral common iliac artery aneurysms. A CT scan of the abdomen showed diffuse, nonatherosclerotic arterial disease with aneurysmal dilatation of the abdominal aorta, the common iliac arteries, and the superior mesenteric artery (Fig. 3). A focal dissection flap was present in the superior mesenteric artery. Echocardiogram showed an ejection fraction of 25%. A coronary CT angiogram revealed an aneurysm with circumferential mural thickening and contrast enhancement of the left main, left anterior descending, left circumflex, and right coronary arteries (Fig. 4). There were also focal, severe segmental stenoses (>70%) without significant plaque involving the left anterior descending, the first and second diagonal, and the ostial circumflex arteries.

Kidney biopsy revealed a combination of membranous nephropathy and chronic active interstitial nephritis with IgG4-dominant immune deposits along both the tubular and glomerular basement membranes. More than 20 IgG4-positive plasma cells/hpf were present within the interstitium. These features were diagnostic of IgG4-related kidney disease with both tubulointerstitial nephritis and membranous glomerulopathy. The kidney injury ultimately resulted in end-stage renal disease. The serum IgG4 concentration, 1980 mg/dL, was approximately 16 times higher than the upper limit of normal (reference 2–120 mg/dL). Other baseline immunologic parameters are shown in Table 3. He was started on prednisone 60 mg/day.

**Table 3:**
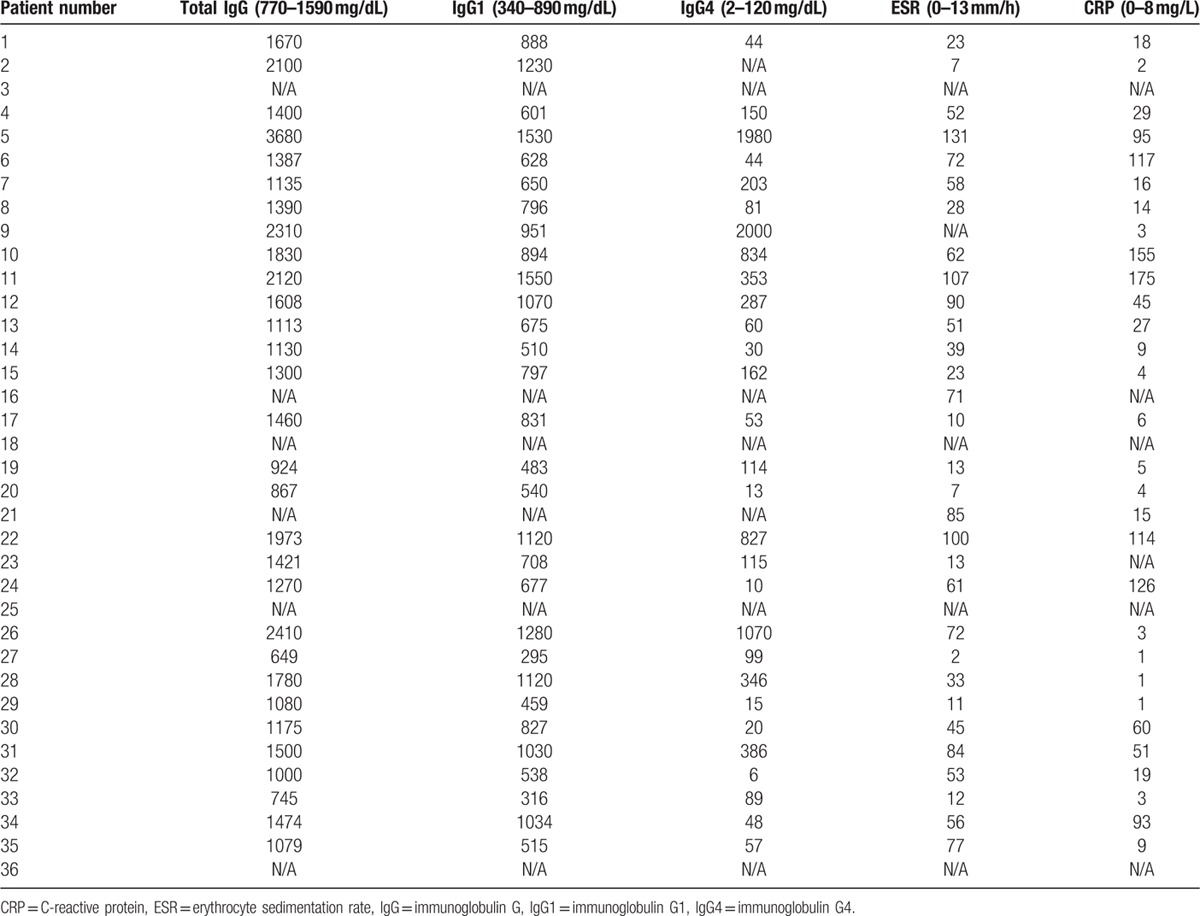
Initial lab values prior to therapy.

One month after beginning treatment, the patient developed unstable angina, prompting urgent coronary artery bypass grafting. Intraoperative findings included grossly visible aneurysmal left main and left anterior descending arteries, a whitish fibrinous discoloration of the ascending aorta without plaque on epiaortic ultrasound, and a whitish discoloration with friability of the left internal mammary artery. The left anterior descending artery was also noted to dissect easily. Operative specimens revealed a lymphocytic infiltrate of the aortic adventitia with scant plasma cells, the majority of which stained for IgG4.

The patient was treated with rituximab (1 g times 2 doses) and was successfully tapered off prednisone in the following 3 months. Serum IgG4 had decreased to 435 mg/dL only 1 month after rituximab treatment, and the serum IgG1, C-reactive protein (CRP), erythrocyte sedimentation rate (ESR), and C3 had all normalized. He was maintained on rituximab (1 g) every 6 months without disease progression or recurrence, but his end-stage renal disease was not reversible.

### 3.4 Patient #7: IgG4-related aortitis and pulmonary artery stenosis

**Figure 5:**
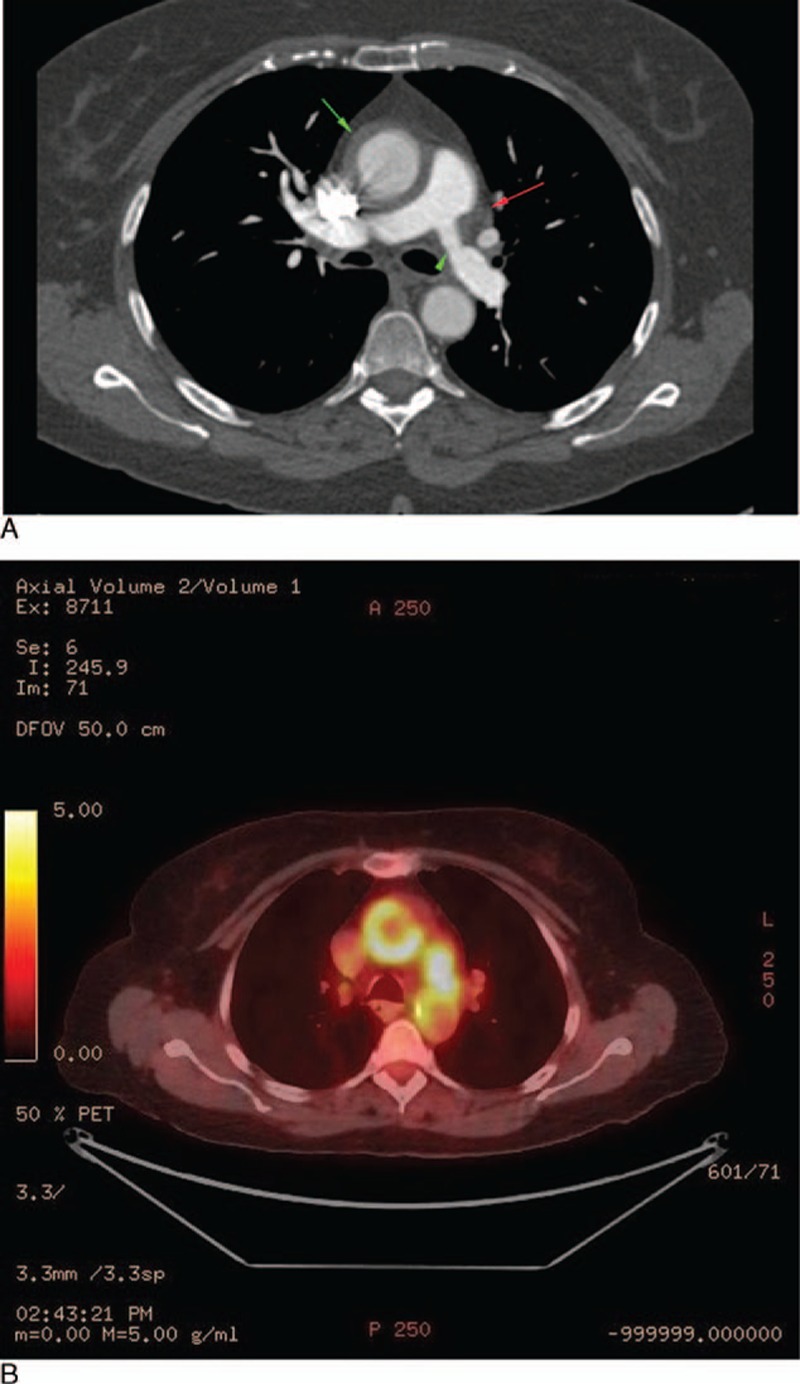
(A) Cross-sectional CT chest with contrast displaying a soft tissue mass compressing the left pulmonary artery (red arrow) with resulting pulmonary arterial stenosis (green arrow head). Also seen is circumferential thickening and contrast enhancement of the ascending thoracic aorta (green arrow). (B) PET-CT showing FDG avidity of both the soft tissue mass around the pulmonary artery as well as the ascending thoracic aortic wall.

A 69-year-old-woman with a history of hypertension, asthma, and seasonal allergies presented with subacute dyspnea on exertion, palpitations, and vocal hoarseness. Physical examination was notable for a systolic bruit over the manubrium with radiation to the carotid arteries (right more than left). Echocardiogram revealed a mild left pulmonary artery stenosis with a right ventricular systolic pressure of 44 mm Hg. The cause of the pulmonary artery stenosis and hypertension was a 4 cm × 3 cm soft tissue mass encasing the left pulmonary artery on CT (Fig. 5A). Another soft tissue mass encased the vocal cords and wall thickening of the thoracic aorta were also present. On PET-CTA, both soft tissue masses and aortic wall were FDG-avid (Fig. 5B).

**Figure 6:**
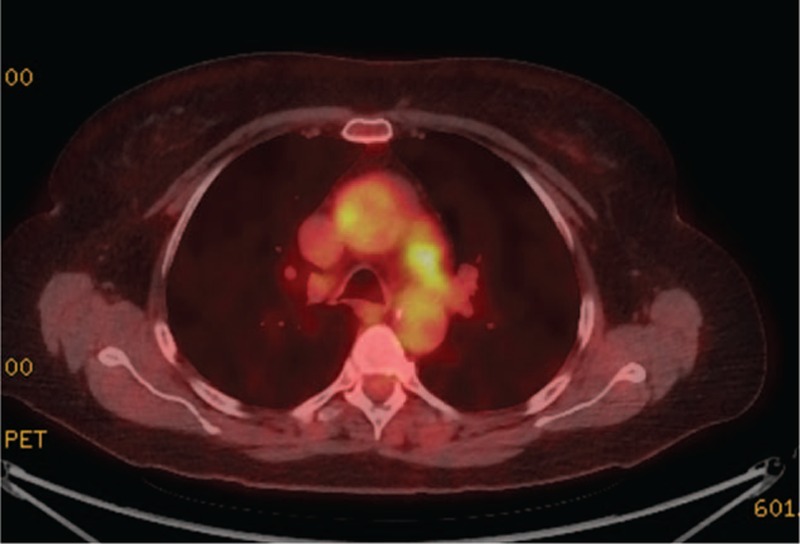
Post-rituximab PET imaging showing decreased FDG avidity of the ascending thoracic aortic wall and soft tissue mass encasing the left pulmonary artery (comparison: Fig. 5B).

The serum IgG4 concentration was elevated at 471 mg/dL (normal 2–120 mg/dL). An assay for antineutrophil cytoplasmic antibodies was negative. The serum IgG4 was 286 mg/dL (2–120 mg/dL), IgE was 1390 IU/mL (0–100), ESR 38 mm/h (0–13 mm/h), and the serum CRP was 16 mg/L (normal <8.0 mg/L). Biopsy of the laryngeal mass demonstrated a lymphoplasmacytic infiltrate, focal areas of storiform fibrosis, scattered eosinophilic infiltrate, >100 IgG4+ plasma cells/hpf, and >40% IgG4+/IgG+ plasma cells. There was no evidence of multinucleated giant cells, granulomatous inflammation, leukocytoclastic vasculitis, or fibrinoid necrosis. She was treated with rituximab (1000 mg × 2 doses) with improvement in symptoms and declines in the serum concentrations of both IgG4 and IgE. Three months after rituximab, a repeat PET scan demonstrated decreased FDG avidity in the areas of previous involvement (Fig. 6). A second rituximab course led to continued decline in these parameters, such that 10 months after the start of treatment her serum IgG4 was 95 mg/dL (2–120 mg/dL), serum IgE was 428 IU/mL (0–100), and acute phase reactants were normal.

### 3.5 Patient #10: IgG4-related aortic dissection

**Figure 7:**
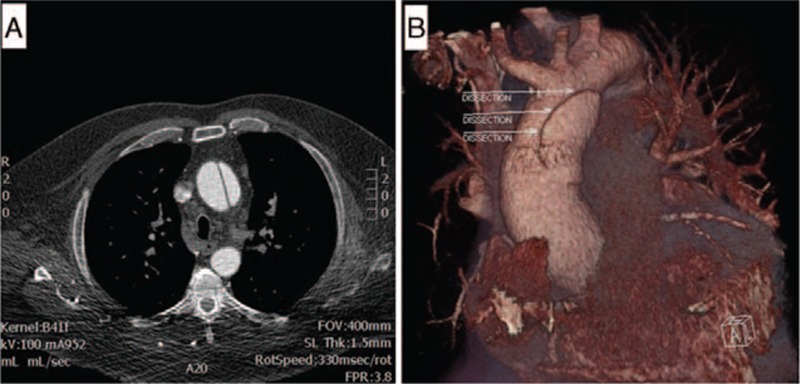
(A) Cross-sectional CT angiography showing a dissection flap in the ascending aorta. (B) 3D reconstruction displaying the same ascending aortic dissection.

A 65-year-old-man with a history of reactive mediastinal lymphadenopathy of unclear etiology was found to have an ascending aortic aneurysm with a focal dissection during preoperative cardiac CT imaging for a planned pulmonary vein isolation procedure (Fig. 7). The patient had multiple medical problems including prior tobacco abuse, chronic obstructive pulmonary disease, congestive heart failure with preserved ejection fraction, atrial fibrillation, hypertension, type 2 diabetes mellitus, obesity, obstructive sleep apnea, stage III chronic kidney disease, and coronary artery disease. He underwent replacement of the ascending aorta and hemiarch, aortic valve replacement, and single-vessel coronary artery bypass grafting. In the operating room, the large ascending aortic aneurysm was found to have a dissection that appeared to be chronic in nature.

Histopathology of the dissection revealed a transmural lymphoplasmacytic infiltrate located predominantly in the media and to a lesser extent in the adventitia and intima. Adventitial fibrosis, nonobliterative phlebitis, occasional eosinophils, and >50% IgG4+/IgG+ plasma cell staining were present in the surgically resected aorta, as well. The cellular infiltrate was limited to the area of the dissection. The serum IgG4 concentration, ESR, and CRP were all markedly elevated at 714 mg/dL (2–120 mg/dL), 73 mm/h (0–13), and 155 mg/L (normal <8.0 mg/L), respectively. CT of the chest and abdomen showed mediastinal and hilar adenopathy, with the largest node measuring 2.4 cm in diameter. An excisional paratracheal lymph node biopsy from 2 years earlier was reviewed with additional immunostaining, revealing an IgG4/IgG ratio >50%.

At follow-up 1 month after surgery, the serum IgG4 was 1148 mg/dL (normal 2–120 mg/dL). He was started on prednisone 40 mg daily and tapered over 9 months. This treatment course was complicated by difficult to control diabetes, weight gain, and pneumonia. Upon tapering the prednisone to 10 mg daily, his serum IgG4 concentration and acute phase reactants began to rise. He was then treated with rituximab (1000 mg × 2 doses). At 2 months, the serum IgG4 concentration had declined from 590 to 363 mg/dL. He experienced another disease flare at 9 months and entered clinical remission with a normal serum IgG4 following a third rituximab course. Over the next several years, the patient was lost to follow-up for extended periods of time because of medical nonadherence. He ultimately died of complications of IgG4-RD following multiple hospitalizations for IgG4-related pleural disease. Autopsy showed active IgG4-RD in multiple organs including the pleura and retroperitoneum. Examination of the aorta demonstrated the infiltration of CD4+SLAMF7+ cytotoxic T cells, recently reported by our group in the context of IgG4-RD.^[17]^

### 3.6 Patient #12: IgG4-related aortitis and arteritis with aneurysm formation

A 74-year-old-man with a history of prostate cancer, RPF causing hydronephrosis, and coronary artery disease presented with chest pain. During the evaluation for angina the patient was found to have an aneurysm of the ascending aorta, descending thoracic aorta, abdominal aorta, and bilateral iliac arteries. The ESR was 140 mm/h (normal range 0–20 mm/h). He ultimately underwent 4-vessel coronary artery bypass and repair of the ascending aortic aneurysm, the pathology of which revealed “nonspecific” aortitis. The patient was treated with a tapering course of prednisone over 1 year, beginning at 40 mg/day. He was referred to our institution for consultation when his acute phase reactants began to rise again.

**Figure 8:**
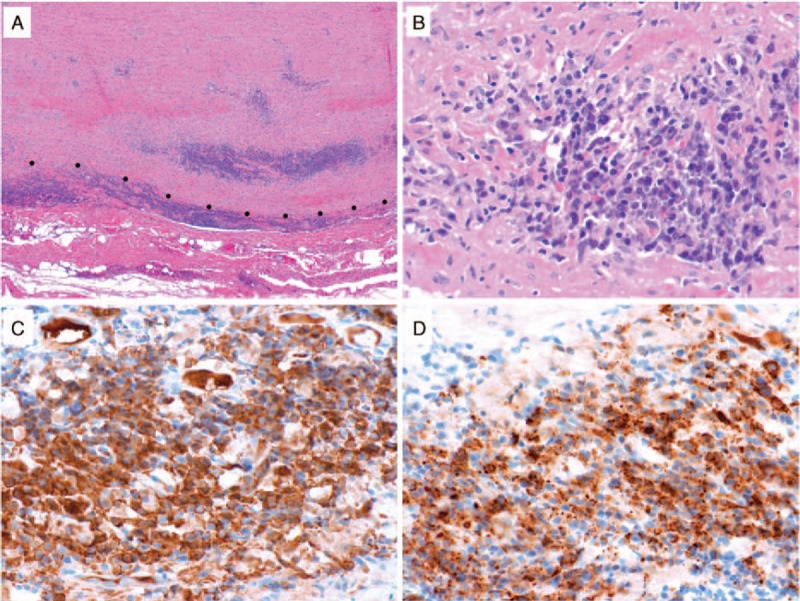
IgG4-related disease in the thoracic aorta. (A) Depicted is a low power (40× magnification) histologic image of the thoracic aorta from Case 12 showing dense inflammatory cell infiltration in adventitia and outer media. The dots indicate the media-adventitia border. (B) At higher power (400× magnification), there is a lymphoplasmacytic infiltrate associated with injury to medial smooth muscle cells. By immunohistochemistry for IgG (C) and IgG4 (D), the majority of the IgG+ plasma cells expressed IgG4 (both 400× magnification).

The patient denied any active symptoms. Physical examination was revealing for bilateral iliac artery bruits. The serum IgG4 concentration was 287 mg/dL (normal 2–120 mg/dL) and the serum IgG1 concentration 1070 mg/dL (340–890 mg/dL). The ESR and CRP were 90 mmg/h (0–13 mm/h) and 45 mg/L (0–8 mg/L), respectively. The patient's blood plasmablast concentration was elevated at 910/mL (normal 1–650/mL). Review of the patient's aorta pathology demonstrated a lymphoplasmacytic infiltrate involving the media and adventitia, storiform fibrosis, and >100 IgG4+ plasma cells/hpf. More than 50% of the IgG+ plasma cells expressed IgG4 (Fig. 8A–D).

He was treated with rituximab because of his disease recurrence after 1 year of prednisone monotherapy. At 3 months’ follow-up, he remained asymptomatic, serum IgG4 decreased to 248 mg/dL, IgG1 to 904 mg/dL, CRP to 29 mg/L, while ESR remained elevated at 83 mm/h. The serum plasmablast concentration decreased to 390/mL and has remained normal. He is being monitored by serial peripheral blood flow cytometry and occasional PET/CT scans.

### 3.7 Patient #35: IgG4-related retroperitoneal fibrosis and secondary periarteritis

**Figure 9:**
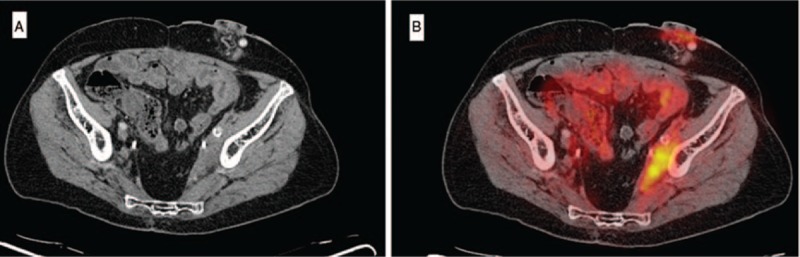
(A) CT pelvis showing a side wall soft tissue mass extending inferiorly and encasing the left iliac vessels and ureter. (B) Associated intense FDG avidity on concurrent PET imaging.

A 65-year-old-man former smoker had a history of colon cancer, for which he had undergone partial colectomy and received chemotherapy and radiation 20 years earlier. He also had a history of asthma, recurrent nephrolithiasis, and hypertension. He presented with acute right flank pain, anuria, nausea and vomiting, and left leg edema on a background of generalized fatigue for 1 to 2 months. Serum creatinine was 2.8 mg/dL (normal 0.8–1.3 mg/dL). Imaging revealed bilateral hydronephrosis secondary to soft tissue thickening along the left pelvic wall as well as multiple, nonobstructive 2 to 3 mm ureteral and kidney stones. Bilateral ureteral stents were placed with resolution of acute kidney injury and symptoms. A PET-CT demonstrated intense FDG avidity of the pelvic wall mass, which extended inferiorly and encased the left iliac vessels and ureter. These findings were concerning for recurrent malignancy (Fig. 9A and B).

A CT-guided core biopsy was performed revealing a lymphoplasmacytic cellular infiltrate with normal flow cytometry and frequent IgG+ plasma cells, the majority of those being IgG4+ on immunohistochemical staining. No malignant cells were identified. These findings were consistent with IgG4-related RPF. The ESR was elevated at 77 mm/h (0–13 mm/h) while the CRP was nearly normal (9 mg/L; normal <8.0 mg/L). The serum IgG4 was normal (57 mg/dL; normal 2–120 mg/dL).

**Figure 10:**
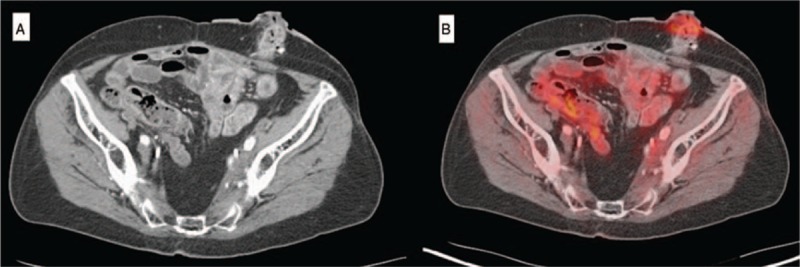
Pos-trituximab imaging revealing an unchanged soft tissue mass on CT (A) but substantially reduced FDG avidity on PET (Comparison Figure 9 A and B).

The patient underwent treatment with rituximab. A follow-up PET/CT demonstrated substantial improvement in the size and FDG uptake of the left pelvic wall mass (Fig. 10A and B). The peripheral blood IgG4+ plasmablast count normalized.

### 3.8 The primary IgG4-related vasculitis and secondary vasculopathy cohort overall

The clinical features of patients with primary IgG4-related vasculitis and secondary vasculopathy are compared in Table 4.

**Table 4:**
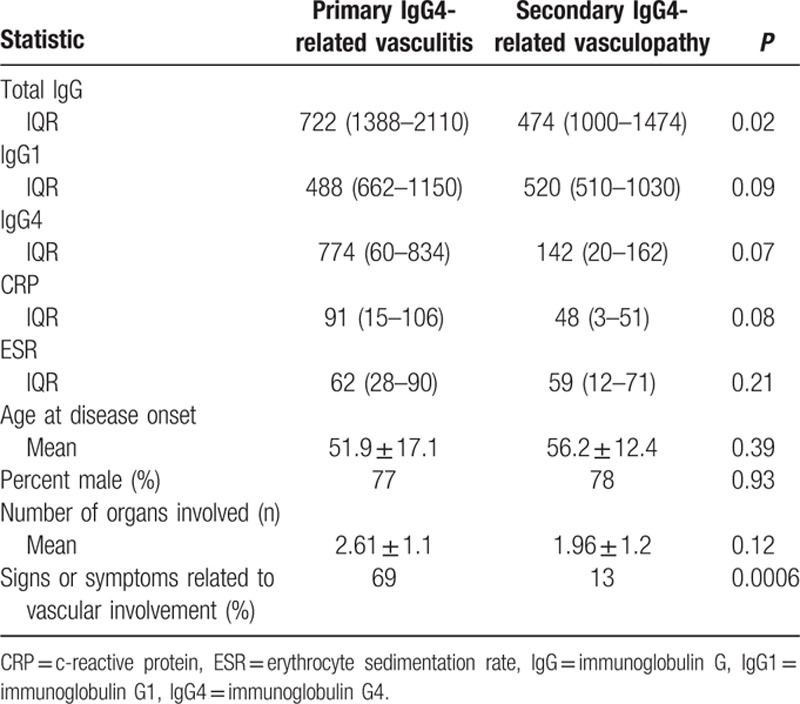
Comparison of clinical features between patients with primary IgG4-related vasculitis and secondary IgG4-related vascular involvement.

#### 3.8.1 Primary IgG4-related vasculitis

Thirteen patients (8% of the entire 160 patient IgG4-RD cohort) had primary involvement of their vasculature by IgG4-RD. Aortitis with or without aneurysmal formation, which affected 9 patients, was observed in the thoracic aorta in 8 patients, in the abdominal aorta in 4 patients, and in both the thoracic and abdominal aorta in 3 patients. Aortitis comprised the most common manifestation among those with primary IgG4-related large-vessel vasculitis, accounting for 69% of this group. Two patients (Patients 4 and 12) had aortic dissections and 1 (Patient 13) had a contained aortic perforation. All 3 of these aortic complications occurred in the context of aneurysm formation. Other manifestations of primary IgG4-related vasculitis included coronary aneurysms (Patients 3 and 5), carotid arteritis (Patients 1, 2, and 8), pulmonary artery aneurysm (Patient 5), pulmonary arteritis (Patient 13), iliac arteritis with aneurysm formation (Patient 12), and iliac phlebitis (Patient 6). These are tabulated in Table 2. Sixty-nine percent of the patients with primary IgG4-related vasculitis presented with signs or symptoms attributable to their vascular involvement. These are listed in Table 1. For example, 1 patient (Patient #2) presented with amaurosis fugax secondary to carotid arteritis. Seven (53%) of the patients with primary IgG4-related vasculitis underwent surgical repair of their affected blood vessels.

**Table 2:**
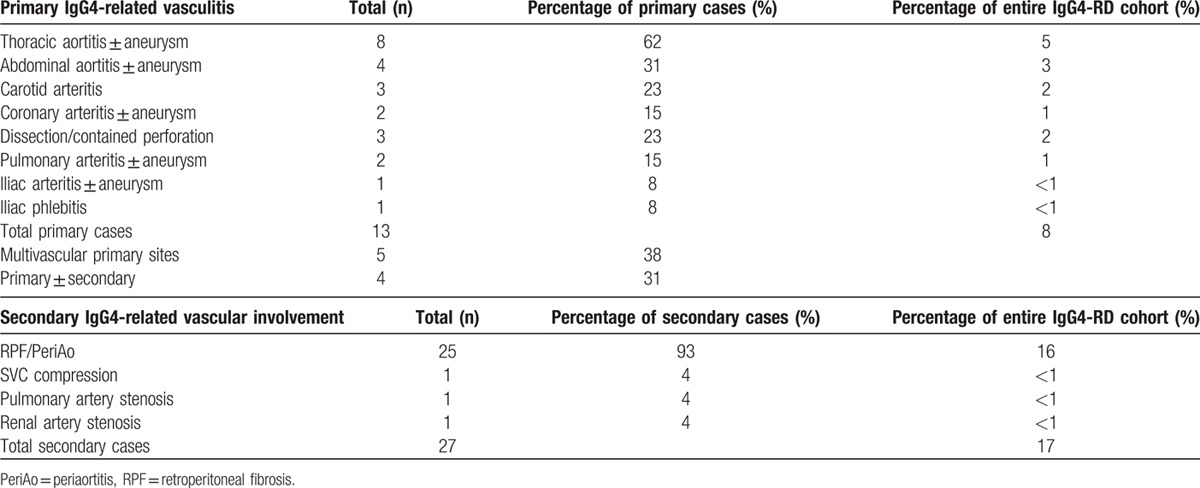
Vascular manifestations of IgG4-RD in this cohort.

#### 3.8.2 Secondary IgG4-related vascular involvement

Periaortitis in the setting of RPF accounted for 27 of 29 patients (93%) of secondary vascular involvement by IgG4-RD. Superior vena cava (SVC) compression (with impending SVC syndrome), renal artery stenosis, and pulmonary artery stenosis, with encasement of the relevant blood vessels by soft tissue masses, accounted for the remaining secondary manifestations. Four patients demonstrated evidence of both primary and secondary vascular involvement, 3 with RPF and 1 with a soft tissue mass encasing the pulmonary artery. Only 13% of patients with secondary vascular involvement presented with signs or symptoms directly attributable to their vascular involvement (Table 1). Flank pain, low back pain, or abdominal pain was the presenting symptom of the majority of patients with RPF.

#### 3.8.3 Extravascular manifestations of IgG4-RD among patients with vascular disease

Of all the patients with IgG4-related vasculitis, 85% (11 of 13) had at least 1 and 54% (7 of 13) had more than 1 extravascular site of involvement. These included the orbit, ear/nose/throat areas, salivary glands, pericardium, lung, pancreas, lymph nodes, bile ducts, kidney, and retroperitoneum. Of the patients with aortitis, 2 of the 9 did not have any extravascular involvement. All patients with secondary vascular involvement had extravascular involvement by definition. RPF accounted for the majority of cases of extravascular involvement, but 11 (48%) had additional organ involvement separate from RPF.

#### 3.8.4 Laboratory features

Laboratory values at presentation and prior to the initiation of treatment are shown in Table 3. Patients with primary IgG4-related vasculitis were more likely to have an elevated total serum IgG compared to those with secondary IgG4-related vascular involvement. Comparison of laboratory features between the 2 groups is displayed in Table 4. Hypocomplementemia was unusual in either group (1 patient in each group). One of these patients had concurrent membranous glomerulonephritis (Patient #5), which has been associated with hypocomplementemia.

### 3.9 Radiologic features

Imaging studies were available on 32 of the 36 patients. Thirty patients (89%) had CT imaging available, of whom 20 (55%) had concomitant PET studies. Based on radiologic findings, the cohort consisted of 8 primary IgG4-related vasculitis patients, 19 secondary IgG4-related vascular involvement patients, and 5 patients with both primary and secondary findings.

A total of 13 patients exhibited primary vascular findings. These findings are summarized in Table 4. Eight patients had primary disease only and 5 had both primary and secondary disease. Those with primary IgG4-related vasculitis did not show a clear predilection for a specific vessel although the thoracic aorta was most often involved. Vascular wall thickening and enhancement with or without aneurysm and perivascular fat stranding were the most common radiologic findings. The epicenter of primary IgG4-related vasculitis was the vessel wall. Aneurysmal dilatation of the thoracic or abdominal aorta was present in eleven patients, aortic dissection in 2 patients, and a contained perforation in one. Luminal narrowing of the aorta was not seen and concomitant atherosclerotic plaque was uncommon.

**Figure 11:**
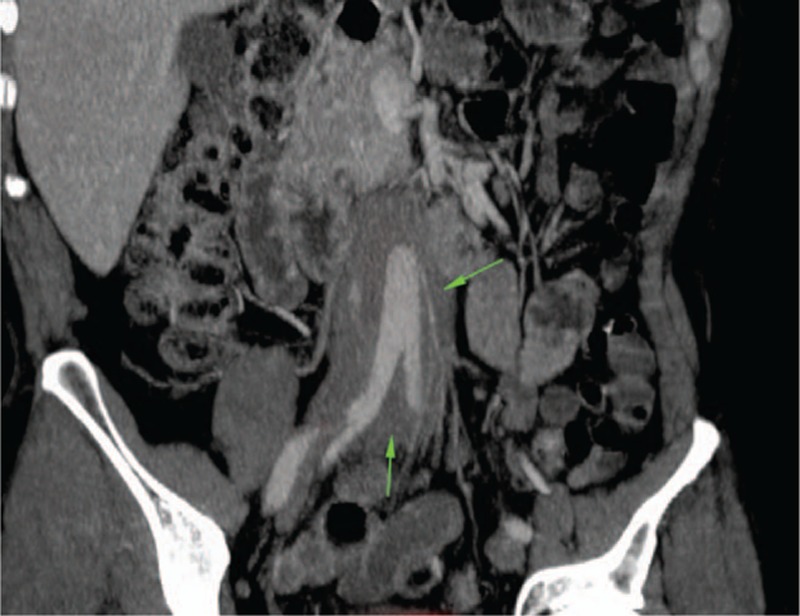
Medialization of the ureters in a patient with IgG4-related retroperitoneal fibrosis (green arrows) and periaortitis.

RPF was the most frequent CT finding among those with secondary IgG4-related vascular involvement. These patients consistently showed the pattern of an infrarenal, periaortic, soft tissue mass, located primarily in an anterolateral distribution and enhancing with contrast. Hydronephrosis was present in nine of the 25 patients (36%) with RPF and ranged in severity from mild to moderate. Two patients required ureteral stenting. Medialization of the ureters was also present in approximately half of patients with periaortitis (Fig. 11). Additionally, encasement of the inferior mesenteric artery and extension to the proximal common iliac arteries were often observed. From the 21 patients with infrarenal periaortitis, 16 (76%) had PET-CT studies, 3 (19%) of whom demonstrated mild FDG uptake, and 13 (81%) of whom showed moderate to significant uptake. Findings on MR angiography were similar to those on CT, demonstrating perivascular soft tissue thickening and enhancement with minimal associated vascular wall enhancement. Brightness was also noted on T2-weighted images. Luminal narrowing was generally not observed.

Three patients demonstrated secondary perivascular disease that did not involve RPF. One of these patients had a soft tissue mass encasing the superior vena cava; the second had a soft tissue mass encasing the left renal artery; and the third (Patient #7) had a soft tissue mass encasing and narrowing the main pulmonary artery. All 3 of these secondary lesions were associated with luminal stenosis of the involved vessel.

### 3.10 Pathology

Diagnostic vascular pathology was available for 5 of the 13 patients with primary IgG4-related vasculitis (Figs. 2 and 8). The pathologic specimens consisted of 4 thoracic aortic resections and one carotid endarterectomy. All patients demonstrated a vasculitis with a lymphoplasmacytic pattern of inflammation. For all 5 patients, there were more than 50 IgG4+ plasma cells per 400× hpf and more than 50% of the IgG+ plasma cells stained for IgG4. The lymphoplasmacytic inflammation involved the adventitia more than the media in the 3 of the 4 aortitis patients, and affected the media more than the adventitia in the fourth. In the carotid endarterectomy, the lymphoplasmacytic inflammation was located in the intimal atherosclerotic plaque. Storiform fibrosis and obliterative adventitial phlebitis were each present in 2 of the 4 aortitis specimens. A modest eosinophil infiltration was present in 1 case of aortitis and in the carotid endarterectomy.

### 3.11 Response to treatment

#### 3.11.1 Primary IgG4-related vasculitis

Ten of the 13 patients with primary IgG4-related vasculitis were treated with rituximab and had adequate follow-up for judgment of response. All 5 of the rituximab-treated patients who were on prednisone at the time of targeted B cell depletion were able to taper completely off of prednisone within 6 months. Of the 4 patients with primary IgG4-related vasculitis who did not undergo surgery *and* had pre- and post-rituximab imaging available, 3 showed radiologic improvement, with reduction of the aortic wall thickening and enhancement (see Table 5). The other patient had stable findings after treatment. In some cases, there was also a decrease in FDG avidity (see Figs. 5B and 6).

**Table 5:**
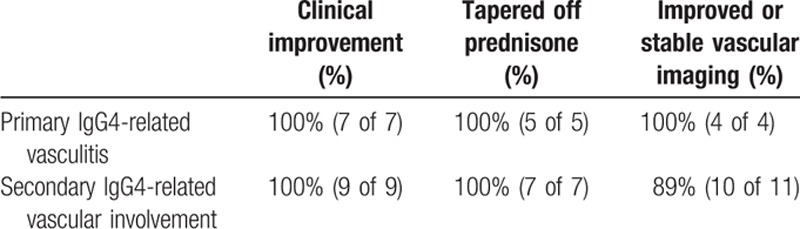
Clinical response to rituximab amongst IgG4-RD patients with vascular involvement.

In total, 7 vascular surgeries were performed in this subgroup. These included 1 carotid endarterectomy, 1 coronary artery bypass graft, and 6 open aortic aneurysm repairs. One of these surgeries (Patient #5) was complicated by friable, dissection-prone vascular tissue of the left internal mammary artery and left anterior descending artery during coronary artery bypass grafting. Only 1 patient was on glucocorticoids preoperatively. The aortic aneurysms in these cases were described as inflamed in appearance with white fibrinous discoloration and sometimes with adherent neighboring tissues. One aortic aneurysm showed intraoperative black areas of necrosis suspicious for impending rupture. All surgeries were successful in correcting the underlying vascular pathology and all of the surgical patients received rituximab following their IgG4-RD diagnosis.

#### 3.11.2 Secondary IgG4-related vascular involvement

Of the 23 patients in this subgroup, 1 was treated with tamoxifen plus prednisone, 6 with prednisone monotherapy, 13 with rituximab, 1 was observed without therapy, and 2 had no information about treatment available. The decision to use tamoxifen plus prednisone in the single patient was made by the referring rheumatologist prior to evaluation in our division. In general, prednisone monotherapy was used as initial treatment. The 7 patients not treated with rituximab responded to initial therapy both symptomatically and radiologically, obviating the need for treatment escalation. Eleven of the 13 secondary patients treated with rituximab had RPF with periaortitis as the vascular manifestation. The decision for rituximab in this subgroup was based on refractoriness to prior therapy (n = 6), baseline contraindication to glucocorticoids (n = 4), or discretion of the treating clinician (n = 3). Of those treated with rituximab, 5 showed radiologic improvement, 5 were unchanged, 1 worsened, and 2 did not have follow-up imaging available. The 1 patient that worsened despite rituximab was a patient with sclerosing mediastinitis and an enlarging mediastinal mass that encircled the SVC. Nine of the 13 rituximab-treated patients showed clinical improvement in their signs or symptoms, 3 were asymptomatic at treatment start date, and no follow-up data were available on 1 patient. Seven of the 8 patients who received rituximab *and* were on prednisone prior to induction therapy were successfully able to taper completely off of prednisone within 6 months of induction. The remaining 1 patient was lost to follow-up.

## 4 Discussion

IgG4-RD has been described in nearly every organ system in the body. Our series demonstrates that large blood vessels are also targeted in this disease and that this large-vessel involvement can be comprised of either a primary IgG4-related vasculitis or a secondary vasculopathy. These disease manifestations sometimes occur in the same patient. The primary vascular manifestations of IgG4-RD described in this series include large-vessel vasculitis of the aorta, pulmonary artery, iliac arteries or iliac veins, as well as a medium-vessel vasculitis involving the carotid or coronary arteries. The arterial beds affected by IgG4-related vasculitis are susceptible to aneurysm formation, which can occasionally be complicated by dissection or perforation. In contrast, arterial stenosis caused by primary IgG4-related vasculitis was not observed in our series, marking an important contrast with other large-vessel vasculitides, particularly giant cell arteritis and Takayasu arteritis. Some of our patients demonstrated aortitis as an isolated organ manifestation, but extravascular involvement typical of IgG4-RD was the norm in cases of primary IgG4-related vasculitis.

The radiologic findings demonstrated in this cohort of patients were distinct from those generally found in atherosclerotic disease. Typical atherosclerotic aneurysm features include visible intravascular mural plaque with or without calcifications; diffuse, multifocal arterial involvement; and minimal if any vascular inflammatory changes in the absence of impending rupture. In contrast, imaging in this cohort demonstrated vessel wall thickening, wall enhancement by gadolinium, FDG uptake on PET studies, and aneurysmal dilatation. Although vascular wall inflammation is a recognized component of the pathogenesis of atherosclerosis both histologically and on PET imaging,^[18]^ the degree of avidity demonstrated in these patients could not be explained by atherosclerotic disease.

The histopathology of large-vessel vasculitis in IgG4-RD has been described.^[5,7,19,21]^ In contrast to the inflammatory infiltrate that centers upon the media in other forms of aortitis (e.g., giant cell arteritis, with its focus upon the internal elastic lamina), the cellular infiltrate of IgG4-RD aortitis predominantly involves the adventitia, with lesser involvement of the media. This distinction has been made clear in a recent consensus statement on the surgical pathology of the aorta with a focus on inflammatory conditions.^[20]^ Similar to the pathology findings in the pancreas, major salivary glands, orbital tissues, and other organs affected by IgG4-RD, the histopathology and immunostaining of vascular involvement demonstrates a lymphoplasmacytic cellular infiltrate, storiform fibrosis, obliterative phlebitis, and an elevated ratio of IgG4/IgG-positive plasma cells.^[5,21,22]^ These findings, which have been documented in both thoracic and abdominal aorta, are similar to those observed in our study. In addition, we describe the findings from a carotid resection with focal IgG4-RD involvement.

The vascular system can also be affected in a secondary manner by the compressive effect of the tumefactive lesions for which IgG4-RD is known. In this cohort, these lesions occurred by far most often in the setting of RPF. IgG4-RD tends to encase the abdominal aorta beginning in the infra-renal region and extending caudally to the iliac arteries. Medialization of the ureters and hydronephrosis are common associated findings, leading frequently to permanent renal injury through postobstructive nephropathy. Patients with secondary IgG4-related vasculopathy appear to be at low risk for aneurysm formation compared to those with primary IgG4-related vasculitis. The abdominal aorta itself appeared to be minimally affected by the surrounding soft tissue in cases of RPF without any cases of aneurysm or stenosis. We did observe 3 cases of arterial narrowing as a result of secondary IgG4-related vasculopathy: one involving the superior vena cava, another the renal artery, and a third, the pulmonary artery.

Perivascular soft tissue abnormalities are minimal in primary IgG4-related vasculitis, a condition in which the inflammation is centered on the vessel wall itself. In contrast, the predominant features of secondary IgG4-related vasculopathy are perivascular soft tissue enhancement and thickening with FDG avidity, but inflammation within the vessel wall itself appears to be minimal, at least as judged by radiology studies.

Clinical differences also exist between these 2 categories of vascular disease in IgG4-RD. Patients with primary IgG4-related vasculitis have greater elevations in total serum IgG and trends toward greater elevations in IgG1, IgG4, and CRP. These patients are also much more likely to present with signs or symptoms directly attributable to the vascular involvement as opposed to those with secondary vasculopathy. Only 13% of patients with secondary vascular involvement presented with signs or symptoms directly attributed to their vascular involvement, a finding consistent with other reports.^[9,23]^ Whereas IgG4-related vasculitis predisposes to aneurysm formation and occasionally to dissection or perforation, secondary IgG4-related vasculopathy is more likely to cause arterial stenosis. The distinction between primary IgG4-related vasculitis and secondary vascular involvement is important in our evolving understanding of this condition, predicting outcomes, and guiding optimal therapy.

Although conclusions from this retrospective study with regard to treatment efficacy are necessarily circumscribed, IgG4-RD patients with vascular involvement appear to respond quite well to B cell depletion. Such treatment was effective for both primary vasculitis and secondary vasculopathy patients alike in regards to clinical improvement, steroid-sparing effect, and radiographic stability. Similar to the findings in this cohort, marked responsiveness of IgG4-RD to B cell depletion was recently published by our group in a prospective clinical trial involving 30 patients with IgG4-RD.^[24]^ Such therapy is particularly beneficial in this aged population often with multiple other comorbidities that are exacerbated by glucocorticoids (e.g., diabetes mellitus, obesity). Seven patients with IgG4-related vasculitis required surgical intervention while the nonsurgical patients treated with rituximab showed stable or improved vascular findings on follow-up imaging. Such findings underscore the importance of early identification and aggressive medical therapy in preventing surgical necessity and poor outcomes. Larger studies of patients with IgG4-related large-vessel disease, both primary and secondary, are required before definitive recommendations regarding treatment can be made.

Our data highlight the substantial frequency of vascular involvement in IgG4-RD, which affected 22.5% of our patient cohort. With nearly a quarter of IgG4-RD patients having some form of vascular involvement, clinicians should maintain a low threshold for obtaining vascular imaging in newly diagnosed patients in the interest of detecting such involvement early and preventing complications. In our experience, PET-CTA with the angiography component including noncontrast, postcontrast arterial and delayed phases is the most appropriate imaging study to evaluate the large vessel walls for evidence of enhancement, thickening, aneurysm, or FDG-uptake. This imaging modality is especially important in distinguishing true vessel wall involvement (primary IgG4-related vasculitis) from adjacent inflammatory tissue (secondary IgG4-related vascular involvement). In the event that vascular involvement is detected on imaging studies in IgG4-RD, the frequency of reimaging must be determined on a case-by-case basis, depending on the nature of the abnormalities found. IgG4-RD is typically an indolent disease by nature, however, and reimaging more frequently than every 6 to 12 months depending on the degree of blood vessel involvement is probably not indicated in most cases.

Our study has both strengths and limitations. We used a large cohort of IgG4-RD patients from a single center. This cohort consisted of a wide variety of organ involvement, reflective of the systemic nature of IgG4-RD. The retrospective nature of this study is a limitation, which carries the potential for recall bias on the signs and symptoms attributable to vascular involvement. In addition, we may have underestimated the true frequency of vascular involvement in our cohort, because the entire cohort of 160 was not evaluated systemically by radiology studies.

### 4.1 Summary

IgG4-RD is a unique and treatable cause of primary large and medium-vessel vasculitis as well as secondary vasculopathy. Although the majority of patients with IgG4-related vasculitis are symptomatic at onset, this is a common disease manifestation with grave consequences that may warrant radiologic screening at the time of diagnosis. IgG4-related vasculitis usually takes the form of an aortitis with aneurysm formation. Periaortitis with relative sparing of the aortic wall is the most common type of secondary vascular involvement. IgG4-related vascular disease is associated with considerable morbidity and even mortality, but patients can be managed effectively if diagnosed early and treated appropriately.
